# Bending Properties of Fiber-Reinforced Composites Retainers Bonded with Spot-Composite Coverage

**DOI:** 10.1155/2017/8469090

**Published:** 2017-10-10

**Authors:** Maria Francesca Sfondrini, Paola Gandini, Paola Tessera, Pekka K. Vallittu, Lippo Lassila, Andrea Scribante

**Affiliations:** ^1^Unit of Orthodontics and Paediatric Dentistry, Section of Dentistry, Department of Clinical, Surgical, Diagnostic and Paediatric Sciences, University of Pavia, Pavia, Italy; ^2^Department of Biomaterial Science and Turku Clinical Biomaterials Centre (TCBC), Institute of Dentistry, University of Turku, Turku, Finland; ^3^City of Turku, Welfare Division, Turku, Finland

## Abstract

Orthodontic and periodontal splints are prepared with round or flat metallic wires. As these devices cannot be used in patients with allergy to metals or with aesthetic demands, fiber-reinforced composite (FRC) retainers have been introduced. Stiffness of FRC materials could reduce physiologic tooth movement. In order to lower rigidity of conventional FRC retainers, a modified construction technique that provided a partial (spot) composite coverage of the fiber has been tested and compared with metallic splints and full-bonded FRCs. Flat (Bond-a-Braid, Reliance Orthodontic Products) and round (Penta-one 0155, Masel Orthodontics) stainless steel splints, conventional FRC splints, and experimental spot-bonded FRC retainers (Everstick Ortho, StickTech) were investigated. The strength to bend the retainers at 0.1 mm deflection and at maximum load was measured with a modified Frasaco model. No significant differences were reported among load values of stainless steel wires and experimental spot-bonded FRC retainers at 0.1 mm deflection. Higher strength values were recoded for conventional full-bonded FRCs. At maximum load no significant differences were reported between metallic splints (flat and round) and experimental spot-bonded FRCs, and no significant differences were reported between spot- and full-bonded FRC splints. These results encourage further tests in order to evaluate clinical applications of experimental spot-bonded FRC retainers.

## 1. Introduction

The retention of teeth in the upper and lower jaw is often required after orthodontic treatment or for periodontal reasons [1]. Usually the stabilization is obtained with flat or round multistranded metallic wires. Splints have been introduced initially as canine-to-canine metallic round retainers [2]. Subsequently, in order to prevent incisors undesirable movements, round and then flat splints bonded to all anterior teeth were introduced [3]. Numerous types of fixed retainers have been described in literature with many sizes, diameters, and shapes [4]. These devices are very common but they cannot be used in patients that have to undergo nuclear magnetic resonance, as during the exam the metal could raise in temperature or interfere with image quality [5]. Moreover, also hypersensitivity to nickel and other metals may cause type IV allergic reactions, thus preventing the use of multistranded splints [6]. Finally, in patients with high aesthetic demands, the presence of metallic structures, even if almost or totally invisible, could slightly lower tooth translucency [7]. On the basis of these concerns, fiber-reinforced composites (FRC) retainers have been introduced for multiple clinical applications [8, 9]. In fact the reinforcement of composite with short or long fibers (Carbon, Aramid, Polyethylene, Glass) provide better mechanical and physical properties over unreinforced materials [10, 11]. FRC splints are metal-free and provide excellent aesthetical results [12].

Clinical reliability of FRC retainers has been tested, showing conflicting results. Some reports reported similar [13–15] or higher [16] efficiency if compared with metallic splints. On the other hand some authors [1] reported less reliability if compared to conventional retainers over time. The variability of the results can be related to different fibers and techniques tested in the various investigations. Therefore it is still unclear if FRCs behavior allows better performances over metallic splints. However FRC retainers are nowadays widely used in clinical dentistry [15].

Moreover these materials showed significantly higher stiffness than conventional metallic splints and wires [17–20]. This characteristic could reduce physiologic tooth movement and this could lead to a higher ankylosis risk for the teeth involved [21].

FRC rigidity is mainly related to composite bulk that covers the entire structure once the fiber is placed onto teeth surfaces [22]. The total composite coverage of FRCs is suggested from the manufacturer [12, 13]. In fact, an experimental preparation technique that would involve composite coverage of FRCs only in correspondence of the teeth would leave the fiber exposed in the interproximal zones, thus mimicking conventional metallic splint rigidity and mechanical behavior.

To our knowledge in literature FRCs have been tested only with a full composite coverage technique, whereas no studies evaluated mechanical properties of FRC splints prepared with a spot-bonded technique. Therefore the purpose of the present report was to evaluate the load to bend FRC splints prepared with both full- and spot-bonding techniques and to compare FRCs with conventional metallic flat or round splints. The null hypothesis of the present report was that there are no significant differences among the various groups tested.

## 2. Materials and Methods

In the present investigation, rectangular metallic splints (Bond-a-Braid, Reliance Orthodontic Products, Bond-a-Braid, Reliance Orthodontic Products Inc., Itasca, IL, USA), round metallic splints (Penta-one 0155, Masel Orthodontics, Carlsbad, CA, USA), and FRCs (Everstick Ortho, StickTech, Turku, Finland) were tested (Table 1).

All materials were divided into coded groups of 10 specimen each (length: 28 mm), according to different bonding techniques:

- FW: flat metallic wire;
- RW: round metallic wire;
- FS: FRC spot-bonded;
- FF: FRC full-bonded.

**Figure 1:**
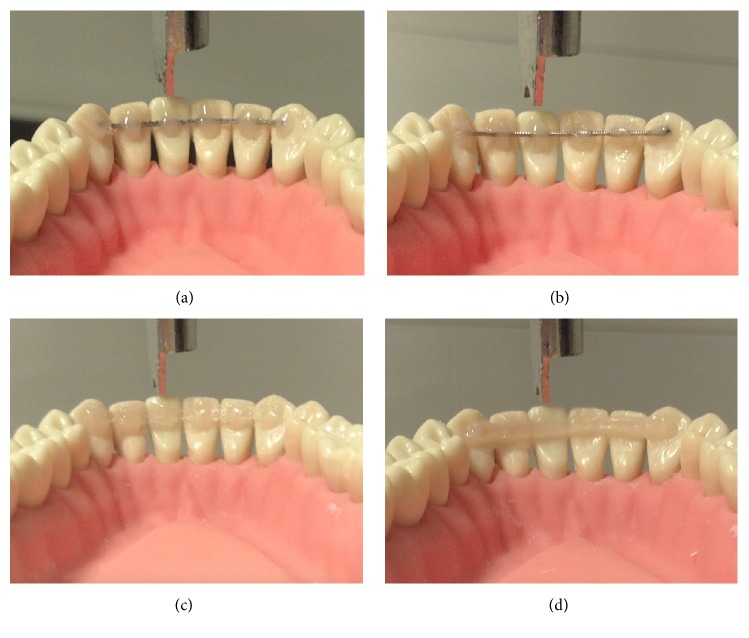
Flat metallic splint (a) round metallic splint (b) experimental spot-bonded FRC (c) and conventional full-bonded FRC (d).

All specimens were then prepared to be bonded to an acrylic Frasaco mandible model, simulating a canine-to-canine splint [23]. Element 3.1 was inserted to the correspondent hole without rigid fixation, thus allowing vertical movement of the tooth. On the other hand, other acrylic teeth were screwed to their correspondent holes. The metallic and FRC splints were bonded to each element from 3.3 to 4.3 with a one-step, self-etch 7th generation bonding agent (G-aenial Bond, GC America, Alsip, IL, USA) and fixed with flow composite (G-aenial Universal Flo, GC America, Alsip, IL, USA). As showed in Figure 1, the composite covered the retainer only in correspondence of each tooth, thus leaving the splint exposed in interproximal zones (Codes FW, RW, and FS). Conversely, composite coverage was performed also in interproximal spaces in full-bonded FRC splints (code: FF). All specimens were then light cured with ahalogen lamp (Elipar S10, 3M, Monrovia, CA, USA) with a light intensity of 1200 mW/cm^2^ and a wavelength range of 430–480 nm for 40 seconds for each tooth.

Subsequently the strength to bend the retainer in correspondence of element 3.1 was measured at 0.1 mm deflection (groups 1 to 4) and at maximum load (groups 5 to 8) with a universal testing machine (Lloyd LRX; Lloyd Instruments, Fareham, United Kingdom). The crosshead speed was set at 1.0 mm per minute [18, 19]. The strength values were recorded in newton with Nexygen MT software (Lloyd Instruments).

Data were submitted to statistical analysis using computer software (R version 3.1.3, R Development Core Team, R Foundation for Statistical Computing, Wien, Austria). Descriptive statistics including mean, standard deviation, minimum, median, and maximum were calculated for the 8 groups. The normality of the data was calculated using the Kolmogorov-Smirnov test and confirmed with graphs in order to avoid misunderstanding due to sample size. An analysis of variance (ANOVA) was used and the repeated measures option was set to adjust for the fact that each specimen gave two outcomes. Tukey test was then applied to evaluate differences among the deflection values of the various groups. Statistical results were adjusted for multiple comparisons. Significance for all statistical tests was predetermined at *P* < 0.05.

## 3. Results

The descriptive statistics (mean, standard deviation, median, minimum, and maximum) of loads (*N*) recorded in the 8 groups are showed in Table 2.

The results of ANOVA indicated the presence of significant differences among the various groups (*P* < 0.001).

**Figure 2:**
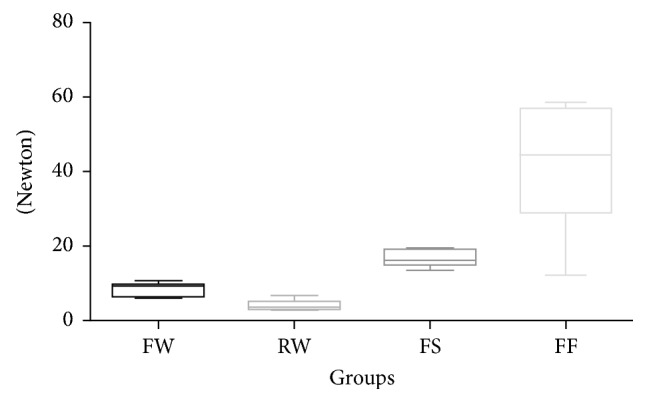
Box plot of strength values (*N*) of the various groups tested at 0.1 mm deflection.

Post hoc test pointed out that, at 0.1 mm (Figure 2, groups 1 to 4) deflection, the lowest flexural strengths were recorded for stainless steel flat (group 1) and round (group 2) wires and for spot-bonded FRC (group 3) retainers that showed no significant differences among them (*P* < 0.05). Significantly higher force levels (*P* < 0.001) were reported for full-bonded FRC splints (group 4).

**Figure 3:**
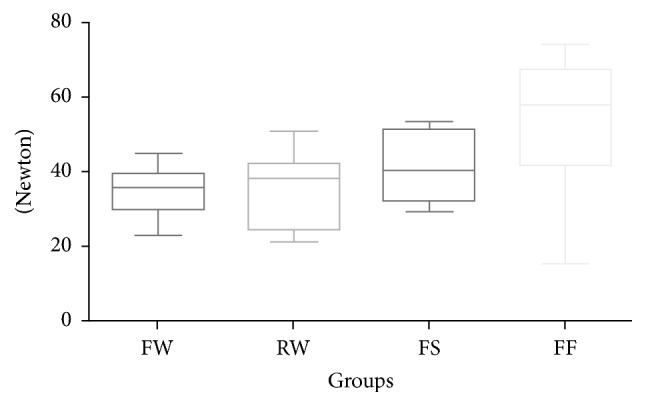
Box plot of strength values (*N*) of the various groups tested at maximum load.

On the other hand, at maximum load (Figure 3, groups 5 to 8) no significant differences (*P* > 0.05) were reported among metallic flat, metallic round, and spot-bonded FRC splints (groups 5, 6, and 7). The highest load values were reported for full-bonded FRC retainers (group 4) that showed no significant differences with spot-bonded FRC splints (*P* > 0.05).

## 4. Discussion

The null hypothesis of the present investigation has been rejected. No significant differences were reported among load values of flat and round stainless steel wires and experimental spot-bonded FRC retainers at 0.1 mm deflection. Significantly higher strength values were recoded for conventional full-bonded FRCs.

At maximum load no significant differences were reported between metallic splints (flat and round) and experimental spot-bonded FRC retainers. Highest strength values were reported with conventional spot-bonded FRCs; no significant differences were reported between spot- and full-bonded FRC splints.

The high bend values of conventional full-bonded FRCs reported in the present investigation is a confirmation of previous reports that showed high rigidity of FRC splints if compared with metallic splints [19, 20] and wires [17, 18]. Even if it is still controversial if the presence of an excessively rigid bonded lingual retainer has a negative effect on the periodontal tissues, ideal retention would allow physiologic tooth micromovements [21]. In fact, clinicians need to splint groups of teeth for many reasons. First of all, after orthodontic treatment the stability is considered one important goal, as the tendency of relapse has been extensively reported, mostly for lower anterior teeth [24]. Moreover, another reason for splinting is related to periodontal problems, as the teeth that have lost part of their supporting bone can be efficiently stabilized with the aid of provisional or definitive retainers connecting them in groups [16]. Finally, splints are widely used after occasional traumas, to stabilize injured teeth [25].

Multistranded wires have been showed to be a successful retention method for over 40 years [2]. They are well accepted from patients and relatively independent of patient cooperation [4], even if a certain number of patients cannot wear these devices. In fact, the release of nickel, chromium, and other metals from brackets, bars, and splints has been demonstrated [26–29]. Moreover, nuclear magnetic resonance exams require the removal of metal appliances in order to avoid heating and image artifacts risks [5]. For these reasons, FRC splints have been introduced. These retainers made of glass fibers nowadays represent the only esthetic and metal-free material, which can be processed in mouth to the desired shape and subsequently can be directly bonded to teeth surfaces [30]. The bonding technique is easy and fast, no laboratory work is needed, and procedures can be completed in a single appointment [13, 14, 18].

FRCs are constituted with continuous unidirectional glass (bundle) fibers in dimethacrylate-polymethylmethacrylate resin matrix as a substructure [30]. The FRCs used for retainer preparation are plain fibers and in prepreg form, so that the fibers were preimpregnated with polymethylmethacrylate from the manufacturer [31].

FRC retainers have been tested in literature and their biomechanical behavior is well known. Previous reports that evaluated FRCs flexural strength reported high load values already at minimum deflections [18, 19]. This is confirmed in the present study for conventional full-bonded FRCs. When comparing the results obtained at 0.1 mm deflection and at maximum load, a significant increase in strength values was reported for flat and round metallic wires and for spot-bonded FRCs. On the other hand, no significant differences were reported for conventional full-bonded FRCs. Therefore, conventional full-bonded FRCs expressed their high rigidity already with minimum deflections, whereas spot-bonded FRCs (as metallic flat and round splints) exhibited high load values only at maximum load. This could be considered an encouraging result improving FRC splints, thus allowing a behavior more similar to metallic retainers than to conventional full-bonded FRC retainers. No other studies to our knowledge evaluated bend values of spot-bonded FRCs.

Other authors evaluated the fatigue of metallic structures and FRCs. Retentive properties of cast metal clasps decrease over time because of metal fatigue. On the other hand, FRC materials showed increased fatigue resistance if compared with metals and may offer a solution to the problem of metal fatigue [31].

Moreover also shear bond strength has been measured both for FRC bundles [32] and nets [33] showing acceptable results. Most common failures in FRC reported in literature include intralaminar matrix cracking, longitudinal matrix splitting, fiber/matrix debonding, fiber pull-out, and fiber fracture, which can be often repaired in patients' mouth [30].

Finally clinical reliability of FRC splints has been reported showing many clinical applications [9, 34] and acceptable failure rates if compared with conventional multistranded metallic wires [13–15]. On the other hand FRC splints present some disadvantages, as higher costs [18], the difficulty to repair if debonded [13], and high rigidity [17]. In fact, when splinting group of teeth excessive rigidity is unwanted from the clinicians as the reduction of physiologic tooth movement could increase ankyloses risk. The low bend values recorded in the present report for experimental spot-bonded technique are promising in reducing FRCs stiffness.

In fact, the conventional FRC construction technique includes enamel etching, washing, and drying. Subsequently a thin layer of adhesive is applied and light cured, and the FRC is placed on teeth surfaces. Finally the a small account of composite paste or flow is used to cover the entire splint and light cured for 40 seconds for each teeth, as suggested from the manufacturer [12–14]. The experimental FRC preparation technique proposed in the present investigation is quite similar. The main difference is that, after FRC positioning, the composite paste or flow that covers the structure has been placed only in correspondence of each tooth, leaving the FRC free of coverage in interproximal spaces. When composites are cured in air, as in clinical practice, an oxygen inhibition layer (0.1 mm thick approximately) is formed on the surface of the freshly cured composite resin. The components of the oxygen inhibition layer present similar composition to those of the uncured resin with reduced amount of photoinitiator [35]. Therefore, also FRCs are affected by this layer, thus reducing their effective diameter of 0.1 mm approximately [36]. The reduction in diameter has been demonstrated to reduce flexural strength of both conventional [17, 18, 37] and nanofilled [38, 39]. This could explain lower bend values showed for experimental spot-bonded FRC in the present investigation. However, in literature no other reports tested spot-bonding technique for FRC retainers. Therefore, further investigation about mechanical characteristics, physical properties, and biocompatibility concerns of these partially uncovered retainers should be conducted before suggesting routinely clinical use.

## 5. Conclusions

The present study demonstrated that experimental spot-bonded FRC showed lower load to bend the retainer at 0.1 mm deflection if compared with conventional full-bonded FRC. Moreover no significant differences were reported among stainless steel flat and round wires and spot-bonded FRCs.

At maximum load no significant differences were reported between metallic splints (flat and round) and experimental spot-bonded FRC retainers, and no significant differences were reported between spot- and full-bonded FRC splints.

The results of the present report encourage further in vitro and in vivo tests in order to evaluate future clinical applications of experimental spot-bonded FRC retainers.

## Figures and Tables

**Table 1:** Materials tested.

Denomination	Flat metallic wire	Round metallic wire	Fiber reinforced composite	Fiber reinforced composite
Code	FW	RW	SF	FF
Manufacturer	Reliance	Masel	StickTech	StickTech
Name	Bond-a-Braid	Penta-one 0155	FRC Ortho	FRC Ortho
Design	Ribbon arch	Coaxial	Unidirectional fiber bundle	Unidirectional fiber bundle
Material	Stainless steel	Stainless steel	E-glass fiber 15 *μ*m	E-glass fiber 15 *μ*m
Dimensions	0.673 mm × 0.268 mm	Diameter: 0.394 mm	Diameter: 0.75 mm	Diameter: 0.75 mm
Unit amount	8 wires	5 wires	1000 fibers	1000 fibers
Bonding technique	Conventional spot	Conventional spot	Experimental spot	Conventional full

**Table 2:** Descriptive statistics (*N*) of load values of the 8 groups tested (each group consisted of 10 specimens).

Group	Code	Material	Shape	Bonding	Deflection	Mean	SD	Min	Mdn	Max	Post hoc
1	FW	Stainless steel	Flat wire	Spot bonded	0.1 mm	8.33	1.68	6.19	8.90	10.76	A
2	RW	Stainless steel	Round wire	Spot bonded	0.1 mm	4.24	1.16	3.23	3.84	6.66	A
3	FS	FRC	Fiber bundle	Spot bonded	0.1 mm	16.67	2.08	13.53	16.18	19.33	A
4	FF	FRC	Fiber bundle	Full bonded	0.1 mm	41.73	16.16	12.18	44.52	58.64	B, C, D
5	FW	Stainless steel	Flat wire	Spot bonded	max load	34.96	6.76	22.86	35.79	44.86	B
6	RW	Stainless steel	Round wire	Spot bonded	max load	35.62	10.26	21.22	38.19	50.74	B
7	FS	FRC	Fiber bundle	Spot bonded	max load	41.44	9.19	29.31	40.32	53.46	B, D
8	FF	FRC	Fiber bundle	Full bonded	max load	52.93	18.84	15.24	57.88	74.12	C, D

Mean with same letters is not significantly different.
